# The mediating roles of cognitive emotion regulation and resilience in the association between life events and sleep quality among medical students

**DOI:** 10.3389/fpsyt.2025.1466138

**Published:** 2025-02-24

**Authors:** Bo Zhu, Xiao-meng Gao, Mei Zhou, Hong-hong Wang

**Affiliations:** ^1^ Xiangya Nursing School, Central South University, Changsha, China; ^2^ Psychology School, Xinxiang Medical University, Xinxiang, China

**Keywords:** life events, sleep quality, resilience, cognitive emotion regulation, medical students

## Abstract

**Background:**

Sleep quality in medical students can be influenced by numerous factors including life events, resilience, and cognitive emotion regulation strategies. Understanding the interplay between these factors is crucial for developing effective interventions to enhance medical students’ mental health and well-being.

**Objective:**

This study examined the association between life events and sleep quality and explored the mediating roles of resilience and cognitive emotion regulation in this relationship.

**Methods:**

This cross-sectional study included 407 medical students from a university in Central Province, China, surveyed between March 15 and March 20, 2023. We employed the Adolescent Self-Rating Life Events Checklist, the Connor-Davidson Resilience Scale, the Cognitive Emotion Regulation Questionnaire, and the Pittsburgh Sleep Quality Index to assess variables. Data analysis involved descriptive statistics, Pearson correlation, and mediation analysis using the SPSS macro Process.

**Results:**

The valid participants (N = 407) were from a medical university with a gender distribution of 29.5% male and 70.5% female. Our findings indicate that higher scores on negative life events significantly predict poorer sleep quality. Furthermore, maladaptive cognitive emotion regulation was also a predictor of poor sleep quality, while resilience was positively associated with beneficial cognitive emotion regulation strategies. Notably, resilience and maladaptive cognitive emotion regulation partially mediated the impact of life events on sleep quality.

**Conclusion:**

The study highlights that life events significantly affect medical students' sleep quality both directly and indirectly through mechanisms involving resilience and cognitive emotion regulation. These insights are vital for framing interventions to improve psychological resilience and adaptive emotion regulation strategies, thereby enhancing sleep quality and overall mental health in medical students. This research contributes to a deeper understanding of how life events impact sleep quality, offering pathways and conditions that could be targeted in future interventions.

## 1 Introduction

Sleep health constitutes a critical global health issue, profoundly influencing individuals' mental and physical health (1, 2). Evidence consistently links high-quality sleep with improved neurocognitive and neuromotor performance, which is particularly crucial for medical students who depend on optimal cognitive functions for heavy academic and clinical practice (3). Despite this importance, extensive research reveals that a significant proportion of the global adult population, including 12.9% to 52.8% of college students in China, suffer from sleep problems (4, 5). This widespread prevalence underscores the urgent need for comprehensive research focused on sleep quality determinants within this demographic.

The relationship between life events and sleep quality has been well-documented. Prior research has shown that stressful life events, such as academic pressure, burnout, emotional exhaustion, and personal mental or health issues, significantly disrupt sleep patterns (6, 7). These life events, particularly during the college years, can result in poor sleep quality, which in turn impacts cognitive functioning and emotional well-being. Understanding how life events impact sleep quality is crucial for improving student health and well-being.

Life events not only affect sleep quality directly, but they may also influence how individuals cope with stress, which can, in turn, impact their sleep. One such coping mechanism is resilience, defined as an individual’s ability to recover from adversity (8). High levels of resilience are associated with better emotional outcomes and improved sleep quality, as resilient individuals are more capable of managing stress and negative emotions (9). Another key coping strategy is cognitive emotion regulation, which refers to the cognitive processes individuals use to manage and modify their emotional responses to stressful situations (10, 11). Cognitive emotion regulation strategies can be divided into adaptive strategies (e.g., reappraisal and acceptance) and maladaptive strategies (e.g., rumination and self-blame). Adaptive strategies are generally linked to better emotional and mental health, while maladaptive strategies tend to exacerbate emotional distress and negatively affect sleep quality (12, 13).

Recent literature has begun to explore the bidirectional relationship between sleep and cognitive emotion regulation. For example, studies have suggested that poor sleep can impair cognitive emotion regulation strategies, leading to increased use of maladaptive strategies such as rumination and self-blame (32, 33). On the other hand, cognitive emotion regulation strategies, particularly maladaptive ones, have been shown to negatively influence sleep quality (34). However, while these associations are documented, the mediating roles of resilience and cognitive emotion regulation strategies in the relationship between life events and sleep quality have not been thoroughly examined.

This study seeks to address this gap by exploring how life events influence sleep quality through the mediating roles of resilience and cognitive emotion regulation strategies. Specifically, we hypothesize that resilience and cognitive emotion regulation strategies mediate the impact of life events on sleep quality. The rationale for this hypothesis is based on the understanding that life events can lead to stress, which in turn affects coping mechanisms (i.e., resilience and cognitive emotion regulation). These mechanisms, whether adaptive or maladaptive, influence sleep quality by shaping emotional and cognitive responses to stress. Thus, exploring how these factors interact is essential to understanding the mechanisms through which life events affect sleep.

The objectives of this study are to provide a deeper understanding of the mechanisms through which life events influence sleep quality and to offer a detailed assessment of the mediating roles of cognitive emotion regulation and resilience. By doing so, this research will contribute valuable insights into effective strategies for enhancing sleep quality among medical students and will propose a conceptual framework for interventions aimed at fostering adaptive coping mechanisms in response to life events.

## 2 Methods

### 2.1 Study design

We employed a cross-sectional study design to examine the relationship between life events and sleep quality and to explore the mediating roles of cognitive emotion regulation and resilience. This design is appropriate for describing associations at a single point in time and allows for an analysis of how variables relate within a specific context. The reporting of this study conforms to the Strengthening the Reporting of Observational Studies in Epidemiology (STROBE) guidelines (13).

### 2.2 Setting and participants

Data collection occurred at a university situated in a Central Province, which represents approximately 10.1% of China's total college student population, thereby situating it among the regions with the highest concentrations of college students in the country. The study targeted medical students, focusing on this group due to prior research indicating that nearly 26% of such individuals suffer from sleep disorders (5).

### 2.3 Sample size

The sample size was calculated using G*Power 3.1 software (14), taking into account a linear multiple regression analysis with 31 predictors. We set the significance level (α) at 0.05 and the effect size at 0.15. According to this, the required sample size was calculated to be 177. However, considering that path analysis typically recommends a minimum sample size of 200 to ensure sufficient statistical power, stability, and reliability of the results, we aimed for a larger sample. In cases where a smaller effect size (0.02) is expected, the required sample size would increase significantly to 539.

In our study, we ultimately collected 423 questionnaires, of which 407 were valid. This larger sample size exceeds the minimum threshold for regression and path analysis, enhancing our findings' robustness and generalizability. The larger sample also ensures that the statistical power remains high, even if the effect size is smaller than anticipated

### 2.4 Recruitment

A questionnaire survey was conducted at one of the medical universities in the province using convenient sampling. In total, 423 questionnaires were distributed among the selected universities, yielding 407 valid responses and an effective response rate of 94.2%.

### 2.5 Inclusion and exclusion criteria

The study has defined clear inclusion and exclusion criteria to ensure the integrity and ethical conduct of the research. Inclusion Criteria: Participants must be registered college students who are actively attending classes at the universities involved in the study. They also voluntarily consent to participate, having understood all aspects of the study. Exclusion Criteria: The study excludes individuals who were unable to understand the study or express themselves effectively for health reasons. These criteria are designed to protect vulnerable individuals and ensure that all participants can adequately engage with the study while providing reliable and informed responses.

## 3 Measurements

### 3.1 Pittsburgh Sleep Quality Index

The Pittsburgh Sleep Quality Index (PSQI, Buysse et al., 1989) (15) was employed to assess sleep quality and disturbances. This instrument comprises 19 items grouped into 7 components: sleep duration, sleep disturbance, sleep latency, daytime dysfunction due to sleepiness, sleep efficiency, overall sleep quality, and usage of sleep medication. Each component is scored from 0 to 3, with the composite scores aggregating to a total range of 0 to 21, where a higher score signifies poorer sleep quality. The PSQI has demonstrated robust reliability and validity in samples of Chinese college students, with a Cronbach's alpha of 0.74 in this study.

### 3.2 Adolescent Self-Rating Life Events Checklist

The Adolescent Self-Rating Life Events Checklist (ASLEC, Liu et al., 1997) (16) quantifies the frequency and impact of negative life events across 6 domains: interpersonal relationships, study pressure, punishment, sense of loss, healthy adaptation, and other factors. The ASLEC features 27 items, each rated on a six-point Likert scale from 0 (did not occur) to 5 (extremely severe), with higher scores indicating greater stress. This scale has shown good internal consistency in this study, with Cronbach's alpha values ranging from 0.78 to 0.92 for the scale and its subscales.

### 3.3 Connor and Davidson Resilience Scale

Resilience was assessed using the Connor and Davidson Resilience Scale (CD-RISC) (17), which includes 25 items across 5 dimensions, reflecting the personal qualities that facilitate thriving in the face of stress and trauma. Items are scored on a 5-point Likert scale from 0 (not true at all) to 4 (true nearly all the time), with higher scores indicating greater resilience. The CD-RISC is widely utilized and validated within research involving Chinese college students, and it showed a Cronbach's alpha of 0.81 in the current study.

### 3.4 Cognitive Emotion Regulation Questionnaire

The Cognitive Emotion Regulation Questionnaire (CERQ, Garnefski & Kraaij, 2006) (18) was used to evaluate cognitive strategies for managing emotions. It identifies 9 strategies, partitioned into adaptive (positive reappraisal, putting into perspective, planning, positive refocusing, and acceptance) and maladaptive (catastrophizing, self-blame, rumination, and other blame). The CERQ consists of 36 items, with responses ranging on a 5-point scale from 1 (almost always) to 5 (almost never). The maladaptive emotion regulation score was created by summing all maladaptive items on the CERQ, with scores ranging from 8 to 40. Higher scores indicated greater use of maladaptive emotion regulation strategies. It has been validated in populations of college students facing adverse events, demonstrating a reliability of 0.79 in this study.

## 4 Ethical considerations

Before initiating data collection, this study received approval from the universities involved and was rigorously reviewed by the Institutional Review Board (IRB) of Xiangya School of Nursing (Protocol Number: E2022110). Detailed explanations regarding the study's objectives and methodologies were provided to all potential participants to ensure informed decision-making. Written informed consent was secured from each participant, outlining the study's background, objectives, and the voluntary nature of their participation. It was explicitly communicated that participants could withdraw from the study at any point without any consequences. To protect confidentiality, no identifiable information was collected during the investigation.

## 5 Data collection

Before data collection, all researchers underwent comprehensive training to ensure thorough familiarity with the questionnaires and a clear understanding of the study's objectives. We gathered 423 responses through a mix of 103 electronic and 320 paper questionnaires. Of these, 16 participants either declined to participate or submitted incomplete questionnaires, resulting in 407 usable responses. This reflects a high response rate of 94.2%.

## 6 Data analysis

Following data collection and thorough verification, the usable data were inputted and analyzed using SPSS version 20.0, along with the PROCESS macro for SPSS. Initially, data screening involved checking for missing values, outliers, and deviations from normality, leading to the exclusion of 16 cases. Subsequently, aggregate scores for each scale were calculated. Pearson correlation analysis was employed to explore relationships between variables. Further, a path analysis was conducted to assess a hypothesized mediation model concerning the impact of various factors on sleep quality. In this model, life events were treated as exogenous explicit variables; resilience and cognitive emotion regulation as endogenous explicit variables; and sleep quality as an endogenous latent variable. Path coefficients were estimated, and model fit was evaluated, providing insights into the interrelations among these variables.

## 7 Results

In this analysis, we scrutinized 407 valid participants’ core sociodemographic attributes. Females predominated the cohort, representing 70.5% (n=287) of the participants, whereas males comprised 19.5% (n=120). Examination of the study year revealed that juniors were the most prevalent, accounting for 39.6% (n=161), closely followed by seniors at 38.1% (n=155). Sophomores and freshmen were less represented, constituting 13.5% (n=55) and 8.8% (n=36), respectively. The vast majority of participants were not only one child, with 83.0% (n=338) reporting siblings, compared to 17.0% (n=69) who were only one child. The geographical origins of participants varied, with 66.8% (n=272) originating from rural environments and 33.2% (n=135) from urban areas.

### 7.1 Correlations among life events, resilience, cognitive emotion regulation, and sleep quality

Our correlational analyses revealed significant relationships among life events, resilience, cognitive emotion regulation strategies, and sleep quality. Specifically, life events positively correlated with Pittsburgh Sleep Quality Index (PSQI) scores, indicating that greater life stress was associated with poorer sleep quality (*r* = 0.279, *p* < 0.01). Moreover, Maladaptive Cognitive Emotion Regulation scores were positively linked to PSQI scores (*r* = 0.221, *p* < 0.01), suggesting that maladaptive emotion regulation strategies contribute to worse sleep quality (**Table 1**).

**Table 1:** Correlations among variables.

Variable	1	2	3	4	5
1. Life Events	1				
2. Resilience	-0.128^*^	1			
3. Maladaptive Cognitive Emotion Regulation	0.368^**^	-0.167^**^	1		
4. Adaptive cognitive Emotion Regulation	-0.100^*^	0.249^**^	—	1	
5. Pittsburgh Sleep Quality Index	0.279^**^	-0.216^**^	0.221^**^	0.072	1

n = 407, **P < 0.01, *P < 0.05.

Conversely, resilience was negatively correlated with PSQI scores (*r* = -0.216, *p* < 0.01), and positively correlated with Adaptive Cognitive Emotion Regulation scores (*r* = 0.249, *p* < 0.01), indicating that higher resilience is associated with both better sleep quality and more adaptive emotion regulation strategies (**Table 1**).

In addition, the Bonferroni p-value correction was applied to the 10 correlation tests. However, due to the stringent threshold set by the Bonferroni correction (*p* < 0.005), none of the correlations reached statistical significance (*p* > 0.005).

### 7.2 Mediating role of resilience and cognitive emotion regulation

In our mediation analyses, resilience was shown to partially mediate the relationship between life events and sleep quality with a mediation effect of 0.103 (*95% CI* [0.220, 0.229]), confirming the protective role of resilience against the adverse effects of life events on sleep. Additionally, maladaptive cognitive emotion regulation strategies also demonstrated a partial mediating effect (*β* = 0.222, *95% CI* [0.063, 0.434]) between life events and sleep quality. To account for multiple correlations in our analysis, we applied a Bonferroni p-value correction. After applying the correction, all p-values remained significant, further supporting the robustness of our findings (**Table 2**).

A chain mediation pathway involving maladaptive cognitive emotion regulation and resilience further elucidated the complex interplay between these variables. This pathway demonstrated a significant mediating effect of 0.279 (*95% CI* [0.098, 0.510]), indicating that maladaptive emotion regulation strategies can impair resilience, subsequently worsening sleep quality. Maladaptive cognitive emotion regulation strategies also demonstrated a partial mediating effect between life events and sleep quality. The mediation analysis revealed that higher life event stressors led to increased use of maladaptive emotion regulation strategies, which, in turn, negatively impacted sleep quality. This indicates that negative cognitive emotion regulation strategies exacerbate the effects of life events on sleep quality (**Table 2**).

**Table 2:** Process analysis.

	Process	Dependent Variable	Independent Variable	*β*	*t*	*P*	*R^2^*	*ΔR^2^*	*F*	p_adj_
Model 1	First Step	PSQI	Life Events	0.279	5.840	0.000	0.078	0.075	34.108	0.000
	Second Step	Resilience	Life Events	-0.128	-2.587	0.010	0.016	0.014	6.693	0.040
	Third Step	PSQI	Life Events Resilience	0.255	5.397	0.000	0.111	0.106	25.182	0.000
				-0.184	-3.882	0.000				0.000
Model 2	First Step	PSQI	Life Events	0.279	5.840	0.000	0.078	0.075	34.108	0.000
	Second Step	Maladaptive Cognitive Emotion Regulation	Life Events	0.368	7.964	0.000	0.135	0.133	63.433	0.000
	Third Step	PSQI	Life Events Maladaptive Cognitive Emotion Regulation	0.137	4.480	0.000	0.094	0.090	20.958	0.000
				0.137	2.698	0.007				0.028

### 7.3 Pathway analyses

Three primary pathways were evaluated for their direct and mediating effects on sleep quality: Pathway 1 (Life Events → Resilience → Sleep Quality): The direct effect of life events on sleep quality was significant (*β* = 1.120, *t* = 5.397, *p* < 0.00, *95% CI* [0.712, 1.528]) with a mediator effect of resilience. The mediation effect of resilience, however, had a relatively small effect size (0.037), suggesting a modest influence of resilience in buffering the impact of life events on sleep quality. The direct effect is significant, but the mediating effect is modest. Furthermore, the Bonferroni correction was applied to the tests, resulting in a direct effect of p = 0.000 and a mediator effect of *p* = 1.374. These findings suggest that the direct effect remains statistically significant (*p* = 0.000), while the mediator effect is no longer significant after the correction (*p-values* > 1) (**Table 3**).

Pathway 2 (Life Events → Maladaptive Cognitive Emotion Regulation → Sleep Quality): This pathway also showed a significant direct effect (*β* = 1.010, *t* = 4.480, *p* < 0.00, *95% CI* [0.562, 1.440]) with a mediator effect of maladaptive cognitive emotion regulation. The effect size for maladaptive emotion regulation was 0.018, indicating a relatively small influence on sleep quality, though still significant. In addition, the Bonferroni correction was applied to the tests, yielding a direct effect of *p* = 0.000 and a mediator effect of *p* = 2.604. This demonstrates that the direct effect remains significant (*p* = 0.000), but the mediator effect is no longer significant following the correction (*p-values* > 1) (**Table 3**).

Pathway 3 (Life Events → Maladaptive Cognitive Emotion Regulation → Resilience → Sleep Quality): Similarly, a significant direct effect was noted (*β* = 0.944, *t* = 4.276, *p* < 0.00, *95% CI* [0.510, 1.278]) with both maladaptive cognitive emotion regulation and resilience as mediators. The indirect effect in this pathway was also relatively small (0.018), suggesting that while maladaptive cognitive emotion regulation and resilience both play a role in mediating the relationship between life events and sleep quality, their combined influence is modest. Moreover, the Bonferroni correction was applied to the tests, with a direct effect of *p* = 0.000 and a mediator effect of *p* = 3.060. This shows that the direct effect persists as significant (*p* = 0.000), while the mediator effect is no longer significant after the correction (*p-values* > 1) (**Table 3**).

The effect sizes for all three pathways are statistically significant, but the mediating effects themselves are relatively small. Specifically, the effect sizes of the mediators—resilience (0.037), maladaptive cognitive emotion regulation (0.018), and their combined influence in Pathway 3 (0.018)—indicate that while these variables significantly mediate the relationship between life events and sleep quality, their practical or real-world impact may be more limited. These findings suggest that while the models are significant, the actual influence of life events on sleep quality through these mediators is not very large in magnitude (**Table 3**).

**Table 3:** Pathway analysis.

Pathway	Direct effect	*t*	*P*	*95% CI*	Mediator effect	Boot SE	*95% CI*	Effect Size
Pathway 1	1.120	5.397	0.000	0.712~1.528	0.103	0.051	0.220~0.229	0.037
Pathway 2	1.010	4.480	0.000	0.562~1.440	0.222	0.094	0.063~0.434	0.018
Pathway 3	0.944	4.276	0.000	0.510~1.278	0.279	0.102	0.098~0.510	0.018

Pathway 1: Life Events → Resilience → Sleep Quality.

Pathway 2: Life Events → Maladaptive Cognitive Emotion Regulation → Sleep Quality.

Pathway 3: Life Events → Maladaptive Cognitive Emotion Regulation → Resilience → Sleep Quality.

## 8 Discussion

This study explored the intricate relationships among life events, sleep quality, resilience, and cognitive emotion regulation in medical students. Notably, we divided cognitive emotion regulation into adaptive and maladaptive types, an approach that is somewhat underrepresented in the existing literature. The findings from our investigation lend robust support to our initial hypotheses: life events negatively influence sleep quality; resilience mediates the relationship between life events and sleep quality; and cognitive emotion regulation pathways significantly impact this relationship.

### 8.1 Correlation analysis of life events, sleep quality, resilience, and cognitive emotion regulation

Our findings align with recent studies demonstrating that negative life events significantly impair sleep quality among college students (12, 19, 20). This correlation is likely mediated by heightened activity in stress-response systems such as the locus coeruleus-norepinephrine (LC-NE) system and the hypothalamic-pituitary-adrenal (HPA) axis, which increase arousal and disrupt sleep (21, 22). These findings are consistent with prior research that suggests life stressors, such as academic pressures in medical school, often lead to poor sleep quality. The relationship between life stressors and sleep quality in our study mirrors these patterns, reinforcing the idea that chronic stress can negatively affect sleep.

Additionally, our data reveal a robust positive correlation between negative life events and the adoption of maladaptive cognitive emotion regulation strategies, exacerbating the adverse effects of stress. This is in line with the work of Gross (35), who highlighted that the use of maladaptive emotion regulation strategies can amplify the negative impact of stress on well-being. Our findings suggest that these maladaptive strategies, such as rumination and catastrophizing, may further elevate the stress response, leading to a vicious cycle that worsens sleep quality. This highlights a critical intervention point—improving the coping mechanisms of students through enhanced support structures and environments could mitigate these maladaptive impacts and improve overall well-being.

In contrast to previous findings (23, 24), our study observed that adaptive cognitive emotion regulation strategies were less utilized than maladaptive ones in the face of adverse events. This suggests the need for targeted interventions to promote more adaptive strategies, such as reappraisal and mindfulness, potentially reducing the psychological toll of stressors on medical students. As Gross (35) and Tugade & Fredrickson (36) suggest, individuals with higher emotional regulation abilities are better equipped to cope with stress, and as such, fostering adaptive strategies could help buffer the effects of stress on sleep quality. Our findings suggest that medical students, in particular, may benefit from interventions that help them better manage their emotions and stressors, especially in the face of academic and personal challenges.

### 8.2 Chain mediating effects of resilience and cognitive emotion regulation

Our study provides a comprehensive examination of how life events influence sleep quality not only directly but also indirectly through the mediating effects of resilience and cognitive emotion regulation. This nuanced understanding underscores the potential of resilience as a mitigating factor against the adverse impacts of stressful life experiences on sleep quality. The selection of resilience and cognitive emotion regulation as mediators is grounded in both theoretical models and empirical research. According to Compas et al. (37), coping mechanisms, such as emotion regulation and resilience, play crucial roles in buffering the effects of stress on well-being. Emotion regulation enables individuals to manage the emotional consequences of life stressors, preventing these emotions from escalating and negatively impacting sleep. Similarly, resilience has been shown to help individuals adapt to adversity, mitigating the negative effects of stress and improving mental health and sleep quality (38). Research within the past five years supports this model, indicating that enhancing resilience through adaptive interpersonal relationships and healthy lifestyle choices, such as regular exercise, can substantially improve psychological well-being and sleep quality (20, 25–27).

Moreover, our findings elaborate on the pathways through which negative life events affect sleep quality. The data suggest that these pathways are modulated by the level of an individual’s resilience, with higher resilience levels potentially diminishing the negative effects of such events, while lower resilience may amplify them. Cognitive emotion regulation strategies play a crucial role in this context, acting as critical mediators. Particularly, the adoption of maladaptive cognitive emotion regulation strategies has been shown to worsen the impact of negative life events on sleep quality (28, 29). While previous studies have demonstrated that the adoption of negative cognitive emotion regulation strategies worsens the impact of life events on sleep quality (30), the current study further expands on this by examining how these strategies mediate the relationship between life stressors and sleep outcomes in a large sample of individuals. This research provides new insights into the mechanisms underlying these associations, highlighting the critical role of negative cognitive emotion regulation strategies in exacerbating the effects of life events on sleep quality. This relationship highlights the need for targeted interventions aimed at fostering more adaptive emotion regulation strategies in response to stress. However, it is important to note that our study did not measure other potential contributing factors such as social support or personality traits, which may also influence both life events and sleep quality. Future research should consider these additional variables to further clarify the mechanisms at play.

Notably, recent literature further delineates the chain mediating effect between these variables. Building on the findings of Zorana et al. (30), who demonstrated that resilience can buffer the impact of stress on sleep by promoting adaptive cognitive emotion regulation strategies, our study extends this work by investigating how individual resilience levels interact with negative cognitive emotion regulation strategies to influence sleep quality in the context of life stressors. This expansion emphasizes the protective role of resilience and adaptive emotion regulation strategies in mitigating the adverse effects of stress on sleep. Similarly, in line with Ning et al. (31), who confirmed a sequential mediation model where life stressors lead to poor sleep outcomes primarily through maladaptive emotion regulation pathways, significantly moderated by individual resilience levels, our study further contributes to this model by testing how negative cognitive emotion regulation strategies mediate the relationship between life events and sleep quality, with resilience moderating this mediation effect. This extension adds to the understanding of the complex interplay between life stressors, emotional regulation, and sleep quality, highlighting the role of resilience in buffering these effects. These findings advocate for a holistic approach in intervention programs that focuses on enhancing resilience and refining emotion regulation techniques to better manage the psychological consequences of life stressors.

## 9 Conclusion

This study represents a novel investigation into the role of resilience and cognitive emotion regulation strategies in mediating the effects of life events on sleep quality, contributing to the growing body of literature on this topic. By dissecting the dual roles of cognitive-emotion regulation strategies—adaptive and maladaptive—our findings offer a novel perspective on how medical students maladaptive the complexities of stress and its repercussions on sleep. It confirms that negative life events significantly worsen sleep quality, as demonstrated by higher Pittsburgh Sleep Quality Index scores, and that this effect is exacerbated by maladaptive cognitive-emotion regulation strategies. Conversely, higher levels of resilience are associated with better sleep quality, highlighting its protective role. This research offers valuable insights into the dynamics of stress and sleep, suggesting that enhancing resilience and promoting adaptive emotion regulation strategies could significantly benefit medical students' sleep and overall well-being.

## 10 Limitations and implications

While our findings contribute valuable insights into the impact of life events on sleep quality through cognitive emotion regulation and resilience, they must be interpreted within the context of certain limitations. A limitation of the current study is the categorization of cognitive emotion regulation strategies as either adaptive or maladaptive. This binary classification may oversimplify the complex interplay of individual strategies and their varying effects on sleep quality. For example, research has suggested that rumination may act as a moderator in the association between stressful life events and sleep quality (Li et al., 2019), and that a finely-tuned balance of strategies may be important in this association (Watershoot et al., 2022). Future studies should consider examining the specific roles of individual strategies or combinations of strategies to provide a more nuanced understanding of these associations. There are also some limitations in the study design such as, firstly, the cross-sectional nature of our data collection restricts our ability to establish causal relationships. We acknowledge that our research cannot infer causality due to the cross-sectional design, and suggest that future research would benefit from longitudinal designs to clarify the directionality of these associations. Secondly, the reliance on self-reported data raises the possibility of common method bias. Although our analyses suggest that this did not significantly skew our results, incorporating multiple data sources and methodological approaches in future studies could enhance the validity of these findings. Additionally, the study sample is limited to medical students from a specific medical university in China, which may affect the generalizability of our results. Finally, we also acknowledge that social support or personality traits, as well as unmeasured variables such as age and other socio-demographic variables, may influence the relationships observed in our questionnaire.

Despite these limitations, our study underscores the significant role that life events play in influencing sleep quality and highlights several practical implications for mitigating sleep-related risks. Recognizing the profound impact of stressors, it is crucial for parents and educators to monitor and address the stress levels in students actively. Implementing stress regulation strategies could foster a more supportive environment, thereby improving sleep quality.

Overall, this study not only advances our understanding of the complex interactions between life events, emotional regulation, resilience, and sleep quality but also provides a foundation for targeted interventions designed to improve the well-being of medical students facing significant life stressors.

## Data Availability

In line with our commitment to transparency and open science, the anonymized data and analysis scripts used in this study are available for public access at https://figshare.com/account/home (DOI: 10.6084/m9.figshare.28159625), ensuring that others can replicate and build upon our findings.
